# SERS-Based Liquid Biopsy of Gastrointestinal Tumors Using a Portable Raman Device Operating in a Clinical Environment

**DOI:** 10.3390/jcm9010212

**Published:** 2020-01-13

**Authors:** Lucretia Avram, Stefania D. Iancu, Andrei Stefancu, Vlad Moisoiu, Alia Colnita, Daniel Marconi, Valer Donca, Elena Buzdugan, Rares Craciun, Nicolae Leopold, Nicolae Crisan, Ioan Coman, Dana Crisan

**Affiliations:** 1 Faculty of Medicine, Iuliu Hatieganu University of Medicine and Pharmacy, 400012 Cluj-Napoca, Romania; avram.lucretia9@gmail.com (L.A.); buzelena@yahoo.com (E.B.); drnicolaecrisan@gmail.com (N.C.); jcoman@yahoo.com (I.C.); crisan.dc@gmail.com (D.C.); 2 Clinical Municipal Hospital, 400139 Cluj-Napoca, Romania; rarescraciun@ymail.com; 3 Faculty of Physics, Babeș-Bolyai University, 400084 Cluj-Napoca, Romania; stefania.iancu22@yahoo.ro (S.D.I.); stefancu.andrei16@gmail.com (A.S.); vlad.moisoiu@gmail.com (V.M.); 4 MEDFUTURE Research Center for Advanced Medicine, Iuliu Hatieganu University of Medicine & Pharmacy, 400349 Cluj-Napoca, Romania; 5 National Institute for Research and Development of Isotopic and Molecular Technologies, 400293 Cluj-Napoca, Romania; alia.colnita@itim-cj.ro (A.C.); daniel.marconi@itim-cj.ro (D.M.)

**Keywords:** gastrointestinal tumors, inflammatory markers, liquid biopsy, principal component analysis-quadratic discriminant analysis, portable Raman spectrometer, surface-enhanced Raman scattering

## Abstract

Early diagnosis based on screening is recognized as one of the most efficient ways of mitigating cancer-associated morbidity and mortality. Therefore, reliable but cost-effective methodologies are needed. By using a portable Raman spectrometer, a small and easily transportable instrument, the needs of modern diagnosis in terms of rapidity, ease of use and flexibility are met. In this study, we analyzed the diagnostic accuracy yielded by the surface-enhanced Raman scattering (SERS)-based profiling of serum, performed with a portable Raman device operating in a real-life hospital environment, in the case of 53 patients with gastrointestinal tumors and 25 control subjects. The SERS spectra of serum displayed intense bands attributed to carotenoids and purine metabolites such as uric acid, xanthine and hypoxanthine, with different intensities between the cancer and control groups. Based on principal component analysis-quadratic discriminant analysis (PCA-QDA), the cancer and control groups were classified with an accuracy of 76.92%. By combining SERS spectra with general inflammatory markers such as C-reactive protein levels, neutrophil counts, platelet counts and hemoglobin levels, the discrimination accuracy was increased to 83.33%. This study highlights the potential of SERS-based liquid biopsy for the point-of-care diagnosis of gastrointestinal tumors using a portable Raman device operating in a clinical setting.

## 1. Introduction

Despite tremendous advancement in our understanding of the molecular mechanism behind the onset and progression of cancer, malignant diseases represent the second cause of mortality in developed countries [1]. Moreover, cancer is expected to become the leading cause of mortality by 2020 in the USA, superseding cardiovascular mortality [1]. While early forms of cancer benefit from a broad range of treatment modalities such as surgical excision, radiation therapy and chemotherapy, there is a lack of efficient treatment for advanced forms of cancer [2]. Consequently, early diagnosis based on screening is recognized as one of the most efficient ways of mitigating cancer-associated morbidity and mortality.

For the moment, high-quality evidence in support of screening in the general population exists only for breast cancer [3], colorectal cancer [4] and cervical cancer [5]. In each case however, the gold standard screening method is represented by an invasive procedure, which limits the proportion of the population willing to participate in screening programs. Consequently, there has been a growing interest towards the development of liquid biopsy as a means of capturing the cancer-associated molecular pattern in biofluids such as plasma or urine [6]. Although most of the liquid biopsy tools rely on genomic, transcriptomic, proteomic or metabolomic profiling, it has been demonstrated that label-free surface-enhanced Raman scattering (SERS) analysis of biofluids is a promising method of identifying patients with cancer in the point-of-care setting [7]. Moreover, by using a portable Raman spectrometer, a small and easily transportable instrument, the needs of modern diagnosis in terms of rapidity, ease of use and flexibility are met.

Raman spectroscopy is a type of vibrational spectroscopy based on the inelastic scattering of laser photons [8]. The potential of this optical technique for several biomedical applications such as the diagnosis of oral cancer [9] has been proved. One of the most important drawbacks of Raman is represented by the low intensity of the signal, which often limits the sensitivity of the method in analyzing biofluids such as serum or urine. However, this feature can be accomplished by amplifying the Raman signal using SERS substrates such as Au or Ag colloids, which display collective oscillations of electrons, termed plasmons [10]. Although both electromagnetic (EM) and chemical effects contribute to the Raman enhancement and the contribution of each mechanism is difficult to pinpoint, we have recently brought compelling evidence showing that the adsorption of analytes onto the nanoparticle’s surface takes place in a specific manner, mediated by single-atom anions or cations [11], in line with the adion-specific adsorption model [12,13].

We have previously demonstrated that SERS analysis of serum captures the metabolic fingerprint associated with breast, colorectal, lung, ovarian and oral cancer, while also enabling a differential diagnosis between these cancer types [14]. We have also showed that the SERS analysis of serum can be combined with prostate specific antigen (PSA) testing for improving the diagnostic accuracy of prostate cancer [15] and that breast cancer can be detected based on the SERS analysis of urine [16]. However, in all previous reports, the SERS analysis was performed using a state-of-the-art Raman spectrometer under ideal laboratory conditions (i.e., dark room), unlike what is realistically achievable in a hospital environment.

This study represents a continuation of our efforts to advance SERS-based liquid biopsy as a novel cancer diagnostic and screening tool. The aim of this study was to validate the SERS profiling of serum as a cancer screening strategy in the relevant hospital environment. To this end, we employed a portable Raman spectrometer, which was installed in a real-life hospital environment. The SERS profiling of serum was performed in the case of control (Ctrl) subjects and patients with gastrointestinal tumors, namely gastric and colorectal cancer (GCRC). In parallel, we also sought to demonstrate the possibility to combine SERS profiling of serum with general inflammatory markers such as C-reactive protein (CRP) levels and neutrophil counts, as well as platelet counts and hemoglobin levels, with the aim of improving the prediction accuracy of the method.

## 2. Experimental Section

### 2.1. Preparation of Human Serum Samples

The study included a consecutive series of 53 patients with biopsy-proven GCRC, diagnosed in a tertiary referral hospital in Cluj-Napoca, Romania between 2017 and 2019. In addition, 25 healthy Ctrl subjects were included, for comparison and discrimination analysis. The patients were included at the time of diagnosis, before starting the treatment regimen. We set the following exclusion criteria: ongoing infections, chronic inflammatory disease (i.e., auto-immune, inflammatory bowel disease, uncontrolled diabetes, etc.), ongoing hematologic diseases, prior history of cancer, ongoing or recent (3 months) corticosteroid or growth factor treatment, NYHA III or IV heart failure, recent (3 months) surgery for other causes. Patients requiring emergency surgery (i.e., bowel/gastric outlet obstruction, acute bleeding) on admission were also excluded.

From the GCRC group, *n* = 18 were diagnosed with gastric cancer and *n* = 35 with colorectal cancer. Demographic and relevant clinical information regarding the patients and Ctrl subjects is presented in Table S1. From each patient, the following parameters were recorded: C-reactive protein (CRP) levels (using the high-sensitivity CRP method), the neutrophil and platelet counts and hemoglobin levels. All patients provided written informed consent for enrolling in this study. The study was approved by the Institutional Review Board of Iuliu Hațieganu University of Medicine and Pharmacy Cluj-Napoca.

From each patient, 12 mL of blood was collected in glass vials and serum was prepared using a standard protocol. For deproteinization, the serum was mixed with methanol in a 1:9 ratio, centrifuged at 5800× *g* for 5 min, and the supernatant was carefully collected for further analysis.

### 2.2. SERS Measurements of Serum Samples

Silver nanoparticles synthesized by reduction of silver nitrate with hydroxylamine hydrochloride (hya-AgNPs) were employed as SERS substrates [17]. Briefly, 17 mg of AgNO_3_ was dissolved in 90 mL ultrapure water (Millipore, Burlington, MA, USA) under stirring. Separately, 17 mg of hydroxylamine hydrochloride was dissolved in 8.8 mL of ultrapure water and 1.2 mL of NaOH 1% was added. The hydroxylamine solution was added to the AgNO_3_ solution under stirring, and the resulting solution changed its color immediately to dark yellow. The colloidal solution was stored at room temperature. All chemicals were purchased from Sigma-Aldrich (Saint Louis, MO, USA). The nanoparticles were characterized by UV-Vis spectroscopy (V-630 Spectrometer, Jasco, Pfungstadt, Germany) (Figure S1).

By pipetting, 45 μL of hya-AgNPs was mixed with 5 μL of deproteinized serum and 5 μL of Ca(NO_3_)_2_ 10 mM. The Ca^2+^ cations adsorb onto the nanoparticle’s silver surface, forming Ca^2+^ adions that promote the chemisorption of negatively charged species, as described by the adion-specific adsorption model [11].

**Figure 1:**
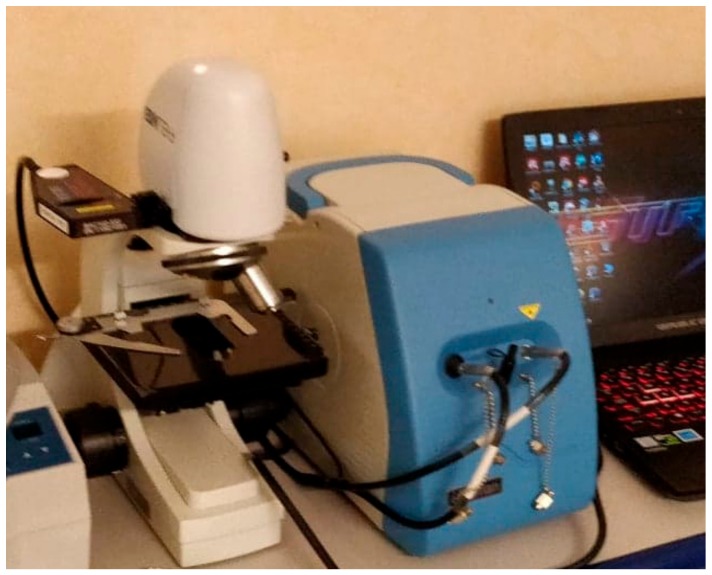
The Raman setup used in this study. The picture shows the portable Raman spectrometer coupled with a microscope.

A drop of 5 μL from this mixture was deposited onto an aluminum foil-covered microscope slide and then analyzed using a portable Raman spectroscope (iRaman 532 nm, B&W Tek, Lübeck, Germany) coupled to a microscope (B&W Tek), the setup being shown in Figure 1.

The spectral resolution of the spectrometer was 4.5 cm^−1^. The 532 nm laser (50 mW) was focused onto the sample through a 20× objective (NA = 0.4), and 4 spectra with 10 s exposure time each were averaged for each acquisition. The laser intensity was set to 30% of the total intensity to avoid the detector saturation. The spectra acquisition was performed in a clinical environment (i.e., without controlling the ambient light conditions). Before each measurement, a reference spectrum was taken as background, which was then automatically subtracted from the final spectrum.

All SERS spectra were recorded randomly in the time range of two days. The spectra were preprocessed by linear baseline subtraction and mean normalization.

### 2.3. Statistical Analysis

Although principal component analysis-linear discriminant analysis (PCA-LDA) is widely used as a classification method, in this study we employed principal component analysis-quadratic discriminant analysis (PCA-QDA), since the variances of the datasets of the two classes were statistically different [18]. The variance comparison test for the inflammatory markers and SERS spectral data (Table S2) was performed using Levene’s test [19]. In the case of inflammatory markers, the variance of the data from each class, GCRC and Ctrl, was found to be statistically different (*p* < 0.05). To check the variance of the spectral data of each class, we first performed PCA on the SERS spectra to reduce the number of variables and to keep only the relevant SERS spectral information. The Levene’s test was performed on the score values resulting from the first 7 principal components (PCs). For PCA-QDA, the first 7 PCs were used as input, which together explained 93.33% of the variance in the dataset. The number of PCs was chosen based on the presence of spectral features in the loading plots of the PCs (Figure S2). All the analyzed serum samples were included in the statistical analysis, without excluding any sample as an outlier.

PCA is an unsupervised multivariate technique, which reduces the dimensionality of the dataset while minimizing the informational loss. The output of PCA is represented by a set of scores (score plots) and corresponding principal component vectors (vector plots), which highlight the SERS bands that have a significant variation in intensity across the dataset. On the other hand, PCA-QDA is a supervised classification method. For each sample, PCA-QDA yields a discriminant value associated with each class (group), and the sample is assigned to the class having the highest discriminant value.

The PCA-QDA model was validated by randomly splitting the samples into a training set with 55 samples, while the remaining 23 samples formed the validation set. The division into training and validation set was repeated 20 times, each time using a random selection of samples. The classification ability is obtained by taking the average diagnostic accuracy for the 20 divisions [20].

Before analysis, the blood tests used in this study, namely, CRP levels, neutrophil counts, platelet counts and hemoglobin levels, were centered to their median values and scaled to ranges. In order to build a statistical model with equal weight on the spectral information (namely 1300 SERS wavenumbers in the 400–1700 cm^−1^ range) and on the blood tests, the weight for each wavenumber from the SERS spectra was set to 1, while the weight for each blood test was set to 325.

The multivariate statistical analysis (PCA-QDA) and the variance comparison tests (Levene’s test) were performed using The Unscrambler X (Camo Software, Oslo, Norway). Univariate analysis was performed using Prism 8 (GraphPad, San Diego, CA, USA). The threshold for statistical significance was set to 0.05.

## 3. Results

### 3.1. Discrimination between the GCRC and Ctrl Groups Based on SERS Data

**Figure 2:**
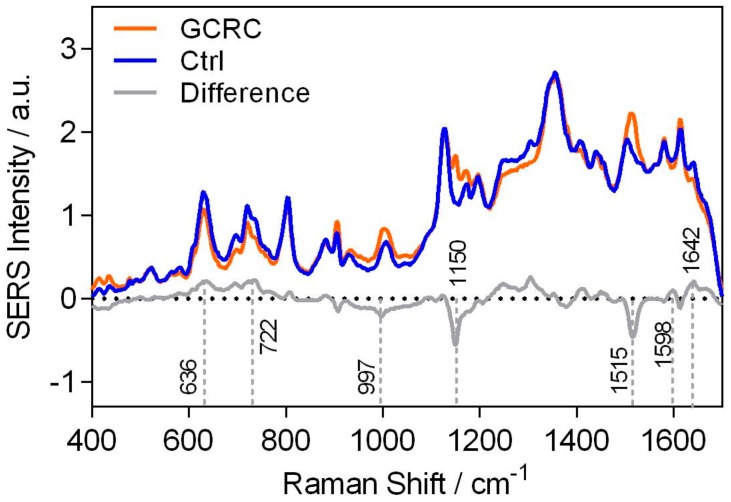
Mean serum surface-enhanced Raman scattering (SERS) spectra of the control group (Ctrl), of the gastric and colorectal cancer (GCRC) group, and their difference spectrum. The SERS spectra of serum were acquired from 53 patients in the GCRC group and 25 control subjects in the Ctrl group.

The average SERS spectra of the Ctrl and GCRC groups, along with their difference (GCRC spectrum subtracted from the Ctrl spectrum), are shown in Figure 2.

The difference SERS spectrum is dominated by several bands, which were attributed to purine metabolites such as uric acid (636,907 and 1642 cm^−1^), xanthine and hypoxanthine (722 and 1598 cm^−1^) and carotenoids (1150 and 1515 cm^−1^) [21].

**Figure 3:**
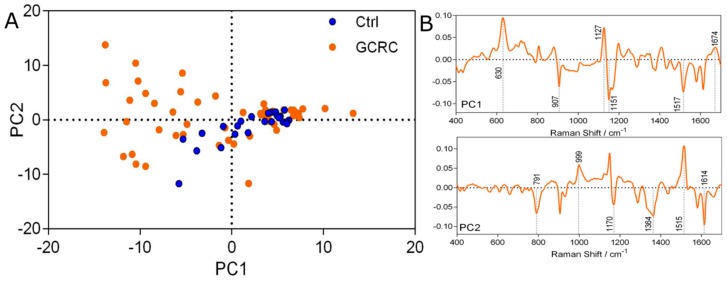
Principal component analysis (PCA) of the SERS spectra of serum. (**A**) The first two principal components (PCs) of PCA shows the clustering of the SERS spectra of serum from 25 control subjects (Ctrl) and 53 patients with gastric and colorectal cancer (GCRC). (**B**) The corresponding loading plots of PC 1 and PC 2.

The score plot of the PCA is depicted in Figure 3, showing the unsupervised clustering tendency of the Ctrl and GCRC groups based on the first two PCs (Figure 3A).

In our study, we obtained a high degree of molecular variability in the serum of the GCRC group, as revealed by the high dispersity of the GCRC samples on the PCA score plot (Figure 3A). This feature of the GCRC samples cannot be attributed to the treatment regimen since patients were treatment-naive at the time when the samples were collected. This finding is in accordance with previous metabolomic studies of serum, hinting at the molecular heterogeneity of cancer [22,23]. Despite being non-specific, this trait could prove useful as an early detection marker, selecting cases for further analysis. The same feature of the two groups can be seen also from their variance (Table S3).

The loading plots of PCA (Figure 3B) show that the SERS bands that contribute the most to the visual separation of the two classes (Figure 3A) are the same SERS bands that appear in the difference spectra (Figure 2). The first two PCs are dominated by the characteristic SERS bands of carotenoids (1151, 1517 cm^−1^), uric acid (630, 907, 999, 1364 and 1674 cm^−1^), and xanthine and hypoxanthine (1127 cm^−1^).

**Figure 4:**
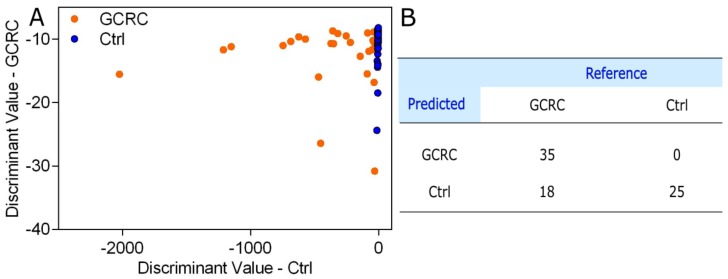
Principal component analysis-quadratic discriminant analysis (PCA-QDA) of the SERS spectra of serum. (**A**) The prediction plot of the PCA-QDA based on the SERS profiling of serum from 53 patients with gastric and colorectal cancer (GCRC) and 25 control subjects (Ctrl) using the first seven principal components as input. (**B**) The confusion matrix yielded by the PCA-QDA of the SERS spectra of serum.

To assess the accuracy of the SERS profiling of serum in discriminating between GCRC patients and Ctrl, we analyzed the SERS spectra by PCA-QDA, the discrimination result being shown in Figure 4.

The PCA-QDA classification yielded a sensitivity of 66.03% in discriminating between GCRC patients and Ctrl subjects based on the SERS profiling of serum samples, a specificity of 100% and an overall accuracy of 76.92%.

### 3.2. Discrimination between the GCRC and Ctrl Groups Based on Inflammatory Markers

In parallel with the SERS profiling of serum samples, we also analyzed the discrimination accuracies yielded by the following blood tests: CRP, hemoglobin levels, platelet counts, and neutrophil counts (Table S4). The univariate discrimination based on hemoglobin levels (AUC = 0.58), platelet counts (AUC = 0.52) and CRP levels (AUC = 0.59) did not reach statistical significance (*p* > 0.05). However, based on the neutrophil counts, a statistically significant differentiation between the Ctrl and GCRC groups was found (AUC = 0.69, *p* = 0.006).

**Figure 5:**
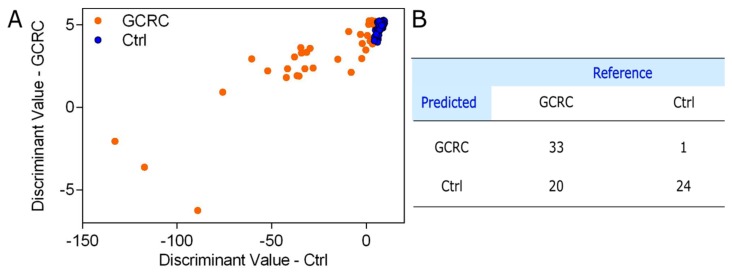
QDA of C-reactive protein (CRP) levels, hemoglobin levels, neutrophil counts and platelet counts. (**A**) The prediction plot of the QDA model combining CRP levels, hemoglobin levels, neutrophil counts and platelet counts from 53 patients with gastric and colorectal cancer (GCRC) and 25 control subjects (Ctrl). (**B**) The confusion matrix yielded by the QDA on CRP levels, hemoglobin levels, neutrophil counts and platelet counts.

Next, we sought to devise a QDA model that combines the four inflammatory markers for discriminating between GCRC and Ctrl groups. The discriminant values of the QDA model are shown in Figure 5A, while the confusion matrix is shown in Figure 5B.

The results demonstrate that the four blood tests (CRP, hemoglobin levels, neutrophil counts and platelet counts) yield a sensitivity of 62.26%, a specificity of 96% and a classification accuracy of 73.08%.

### 3.3. Discrimination between the GCRC and Ctrl Groups Based on SERS Data Combined with Blood Tests

**Figure 6:**
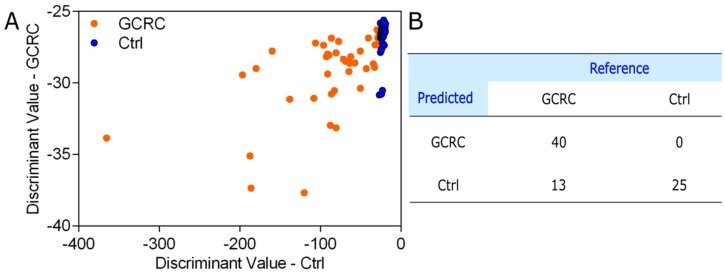
PCA-QDA based on SERS spectra of serum and blood tests. (**A**) The prediction plot of the PCA-QDA model combining CRP levels, hemoglobin levels, neutrophil counts and platelet counts with the SERS profiling of serum (1300 wavenumbers for each sample) from 53 patients with gastric and colorectal cancer (GCRC) and 25 control subjects (Ctrl), using the first seven principal components as input. (**B**) The confusion matrix yielded by the PCA-QDA on SERS spectra of serum and blood tests.

In order to improve the discrimination between GCRC and Ctrl, the four blood tests were combined with the SERS spectra (1300 wavenumber values) of the serum. The discriminant values of the PCA-QDA model combining CRP level, neutrophil count, platelet count and hemoglobin level with the SERS profiling of serum are shown in Figure 6A, while the confusion matrix is shown in Figure 6B.

The PCA-QDA results in Figure 6B, based on the combined SERS spectra and blood tests, correspond to a sensitivity of 75.47%, a specificity of 100% and an overall accuracy of 83.33%, which is superior to the overall accuracy yielded by either SERS profiling of serum alone (76.92%) or a combination of the four blood tests (73.08%).

In order to validate the PCA-QDA model, the samples were randomly divided into a training set containing 55 samples, whereas the remaining 23 samples formed the validation set, this procedure being repeated 20 times. Based on the prediction on the 20 validation sets, a sensitivity of 78.18%, a specificity of 82.03% and an overall accuracy of 80.10% were obtained.

### 3.4. PCA-QDA Model for Cancer Grading

Since early diagnosis based on screening is recognized as one of the most efficient ways of mitigating cancer-associated morbidity and mortality, we tested the possibility to attain a stage-specific discrimination between GCRC patients and Ctrl subjects based on the SERS spectra of serum combined with blood tests.

**Figure 7:**
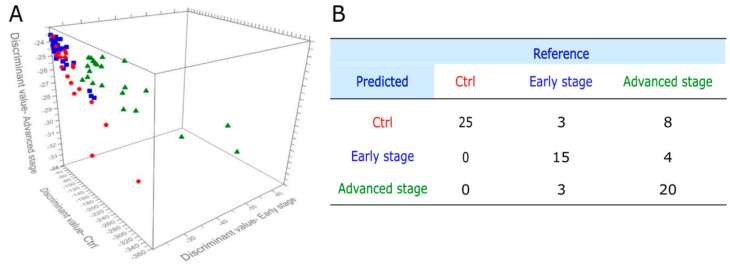
PCA-QDA model for cancer grading, obtained by combining CRP levels, hemoglobin levels, neutrophil counts and platelet counts with the SERS profiling of serum. (**A**) Prediction plot of the PCA-QDA of 25 control subjects (Ctrl), 21 patients with stage I or II gastric and colorectal cancer (early stage), and 32 patients with stage III and IV gastric and colorectal cancer (advanced stage), using the first seven principal components as input. (**B**) The confusion matrix of cancer grading yielded by the PCA-QDA on SERS spectra of serum and blood tests.

The GCRC group was therefore split into early forms of cancer (stage I and II) and advanced stages (III and IV). The prediction plot of the PCA-QDA model, which included SERS spectral data (1300 wavenumbers for each sample), CRP levels, hemoglobin levels, and neutrophil and platelet counts, is shown in Figure 7A, while the corresponding confusion matrix is shown in Figure 7B.

The overall accuracy of the discrimination between Ctrl subjects, early and advanced stage GCRC patients was 76.92%, with a specificity of 100%. The sensitivity of early stage GCRC detection was 71.42%, while the sensitivity for advanced stage GCRC detection was 62.5%. The PCA-QDA statistical model yields better classification accuracy for detecting early stage GCRC (71.42%) compared to advanced stage GCRC (62.5%). This finding is in line with the previous report of Cervo et al. [24], which found better discrimination accuracy for early stage breast cancer samples compared to advanced stage.

## 4. Discussion

The aim of this study was to translate the SERS-based profiling of serum samples for cancer screening from a research-based laboratory into the clinical setting. SERS has the potential to be used as a universal screening method since it is able to capture metabolic differences in the serum. Furthermore, the SERS spectral data can be used in combination with specific blood tests or inflammatory biological markers to improve the diagnostic accuracy.

Inflammation occurs as a consequence of anarchic cell growth, altering the surrounding microenvironment largely due to increased cellular turnover, necrosis, mass-effect and altered local vascularization. Therefore, significant changes can occur with regards to the serum molecular pattern, with indirect markers of malignant processes being increasingly recognized. These specific alterations are especially visible when purine metabolism is analyzed, as purines are essential building blocks for DNA synthesis and cell proliferation. Numerous recent papers have provided an in-depth analysis of purine metabolism in cancer, and our findings are following the same trend in confirming basic scientific results. Consequently, tracing the purine footprint could unlock an important gateway towards early detection of cellular overgrowth [25,26,27].

Acquiring SERS spectra from protein-rich biological fluids such as serum requires the removal of proteins, because these hinder the adsorption of metabolites onto the metal surface by forming a protein corona [28]. In this study, the removal of proteins was performed by precipitating them with methanol, a procedure which has the advantage that it retains carotenoids. Retaining carotenoids in the serum is important since cancer patients are known to display lower carotenoid levels [29]. Moreover, carotenoids exhibit intense Raman spectra when using a 532 nm laser as an excitation source because of the pre-resonance with the electronic transition between molecular orbitals [30]. Another widely used method of removing proteins from serum for SERS profiling is represented by filtration, but this method has the drawback that it also removes carotenoids [24,28].

The SERS profiling of serum was performed using hya-AgNPs activated with Ca^2+^, yielding intense SERS bands from purine metabolites such as uric acid, hypoxanthine and xanthine, which contribute to the differentiation between the GCRC and Ctrl groups. In recent studies, we brought compelling evidence showing that acquiring SERS spectra from anionic compounds, such as purine metabolites, requires the presence of atomic cations such as Ca^2+^ or Mg^2+^, which mediate the specific adsorption of anionic species, whereas anions such as Cl^−^ mediate the specific adsorption of cationic analytes [11,12], in line with the adion-specific adsorption mechanism [13].

The overall accuracy obtained using SERS spectra alone (Figure 4) is in line with our previous studies, confirming that SERS-based screening of malignant pathologies could be used as a reproducible clinical diagnosis method [15,16,31]. For example, we analyzed a group of *n* = 253 cancer and control subjects, from which 109 subjects were patients with colorectal cancer [14]. Using PCA-LDA, we could classify the colorectal patients and the control group with an overall accuracy of 78%, which is similar to the accuracy reported in the current study (76.92%) performed with a portable Raman spectrometer.

We chose CRP levels, neutrophil counts, platelet counts and hemoglobin levels as biological markers of inflammation in cancer because tumor development assumes a state of chronic inflammation, which explains why CRP level and neutrophil count are elevated in oncological patients [32], as it was also proved by the univariate statistical analysis. There are multiple ways by which chronic inflammation promotes hematologic imbalance, especially in the setting of malignant disease. Consequently, altered platelet counts are expected, while anemia is frequently encountered both because of altered hematopoiesis and increased loss via occult or overt gastrointestinal bleeding [33]. The QDA model, which combined these four inflammatory markers, yielded an overall classification accuracy of 73.08%, a sensitivity of 62.26% and a specificity of 96%. Although most GCRC patients display perturbed CRP levels, neutrophil counts, platelet counts and hemoglobin levels, which translates into a high specificity of the QDA model, the perturbations of these parameters are not characteristic for GCRC patients, resulting in low sensitivity.

The PCA-QDA model, which combined the four clinical markers and SERS spectral data (Figure 6), yielded an overall classification accuracy of 83.33%, which is superior to the accuracy yielded by either SERS profiling of serum alone (76.92%) or a combination of the blood tests (73.08%). These results are in line with previous reports from our group showing that the classification accuracy between prostate cancer patients and patients with benign prostate diseases can be improved by combining SERS profiling of serum with prostate specific antigen levels [15]. The possibility of combining SERS-based liquid biopsy with well-established markers of cancer is an interesting strategy that has been insufficiently explored so far.

## 5. Conclusions

The results of this study showed that SERS-based liquid biopsy of patients with digestive tumors can be achieved using a portable Raman device operating in a real-life clinical environment. The classification accuracy yielded by combining SERS analysis of serum with CRP levels, neutrophil counts, platelet counts and hemoglobin levels was superior (accuracy 83.33%) to the classification accuracy yielded by SERS profiling alone (accuracy 76.92%) and to the one yielded by blood tests (accuracy 73.08%). Moreover, based on the blood tests and SERS spectra of serum, a stage-specific discrimination between Ctrl, early stage and advanced stage of GCRC was attained with an accuracy of 76.92%. Further studies are needed to assess the role of point-of-care Raman systems in real-life hospital settings. The sensitivity and specificity of this technique should be continuously compared with current gold standards in cancer diagnosis in order to better understand the efficacy of Raman in screening scenarios and to improve its accuracy, as the theoretical advantages have already been singled out.

## Supplementary Materials

The following are available online at https://www.mdpi.com/2077-0383/9/1/212/s1, Figure S1: The UV-Vis absorbance spectrum of hya-AgNPs. Figure S2: The loading plots corresponding to PCs 3–7, which resulted from the PCA analysis of the SERS spectra of serum samples of *n* = 53 patients with gastrointestinal tumors and of *n* = 25 Ctrl subjects (the loading plots of PC 1 and PC 2 are shown in Figure 3). Table S1: The demographic and clinical information of patients and controls. Table S2: The results of Levene’s test for the first seven PCs and the inflammatory markers. *P*-value < 0.05 shows significant difference between the control and GCRC groups. Table S3: The results of the univariate data analysis for the CRP level, neutrophil count, platelet count and hemoglobin level. Table S4: The descriptive statistics for the first seven PCs and for the inflammatory markers for the two groups, Ctrl and GCRC.
