# Supplementary material for: SERS-Based Liquid Biopsy of Gastrointestinal Tumors Using a Portable Raman Device Operating in a Clinical Environment
Source: J Clin Med. 2020 Jan 13;9(1):212. doi: 10.3390/jcm9010212 (PMC7019591; doi:10.3390/jcm9010212)
Supplement: Supplementary file 1 [file jcm-09-00212-s001.pdf]

Supplementary

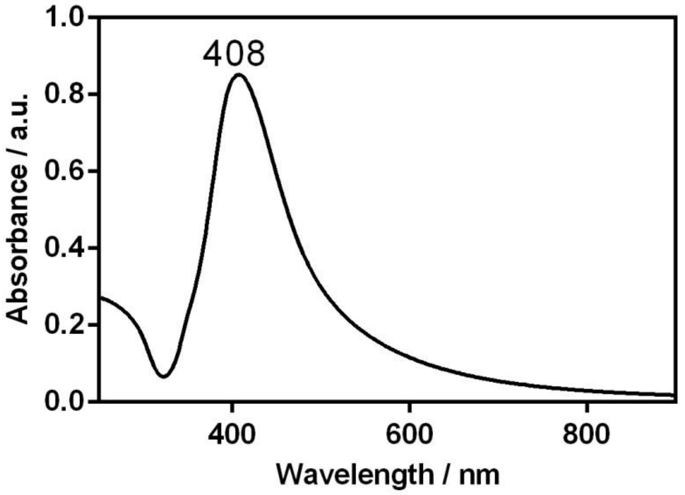

Figure S1. The UV-Vis absorbance spectrum of hya-AgNPs.

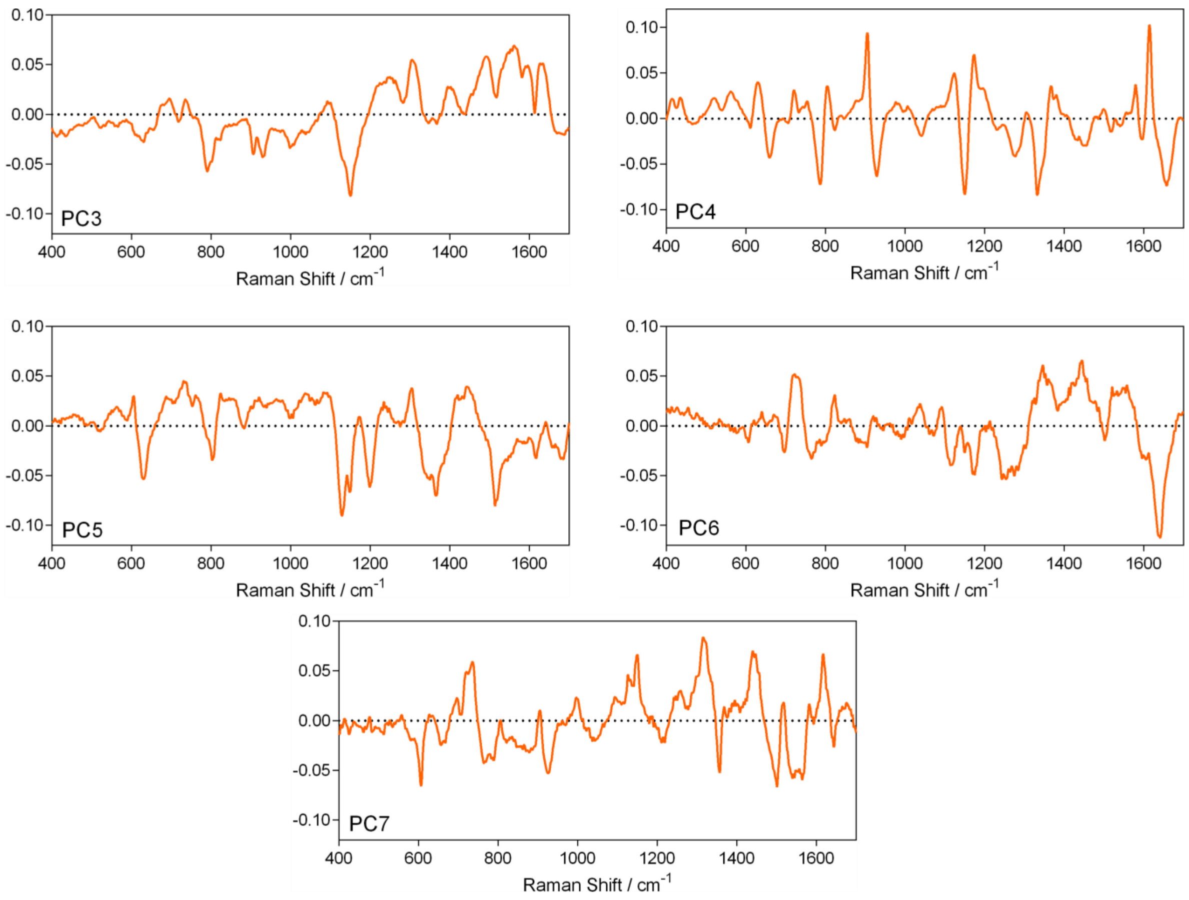

Figure S2. The loading plots corresponding to PCs 3–7, which resulted from the PCA analysis of the SERS spectra of serum samples of n = 53 patients with gastrointestinal tumors and of n = 25 Ctrl subjects (the loading plots of PC 1 and PC 2 are shown in Figure 3).

Table S1. The demographic and clinical information of patients and controls.

Sample	Sex	Age	Type of Tumor	Group (0 = Ctrl, 1 = colorectal cancer, 2 = gastric cancer)	Grade (0 = Ctrl, 1 = early, 2 = advanced)	Height (cm)	Weight (kg)	BMI
Ctrl 1	M	56			0	178	76	23.98
Ctrl 2	F	28			0	176	60	19.36
Ctrl 3	F	46			0	268	88	32.27
Ctrl 4	M	30			0	275	64	20.89
Ctrl 5	M	30			0	270	65	22.49
Ctrl 6	F	30			0	263	52	29.57
Ctrl 7	F	64			0	263	77	28.98
Ctrl 8	F	46			0	260	67	26.27
Ctrl 9	F	30			0	263	65	24.46
Ctrl 10	F	32			0	278	65	20.5
Ctrl 11	M	30			0	274	69	22.79
Ctrl 12	F	30			0	266	60	25.78
Ctrl 13	M	30			0	275	230	42.44
Ctrl 14	F	45			0	256	45	28.49
Ctrl 15	F	35			0	258	65	26.03
Ctrl 16	M	38			0	282	90	27.27
Ctrl 17	F	44			0	262	55	20.95
Ctrl 18	F	30			0	273	62	20.72
Ctrl 19	F	30			0	260	54	22.09
Ctrl 20	F	35			0	262	64	24.69
Ctrl 21	F	35			0	272	68	22.98
Ctrl 22	F	30			0	265	50	28.36
Ctrl 23	M	30			0	276	72	23.24
Ctrl 24	M	35			0	272	70	23.66
Ctrl 25	M	30			0	270	64	22.24
Patient 1	F	83	rectal cancer	1	1	265	60	22.03
Patient 2	F	84	gastric cancer	2	1	268	56	29.84

Patient 3	M	67	sigmoid cancer	1	2	275	68	22.2
Patient 4	F	69	colon cancer	1	2	265	60	22.03
Patient 5	M	72	gastric cancer	2	2	268	70	24.8
Patient 6	F	66	rectal cancer	1	2	265	55	20.2
Patient 7	F	73	gastric cancer	2	2	272	62	20.62
Patient 8	M	43	gastric cancer	2	2	270	80	27.68
Patient 9	F	40	sigma cancer	1	1	263	62	22.33
Patient 10	F	55	sigma cancer	1	2	270	80	27.68
Patient 11	M	77	gastric cancer	2	2	275	60	29.59
Patient 12	M	57	rectal cancer	1	2	278	90	28.4
Patient 13	F	75	colon cancer	1	1	266	70	25.4
Patient 14	M	76	rectosigmoid cancer	1	2	278	70	22.09
Patient 15	F	39	small intestine cancer	1	2	250	56	24.88
Patient 16	F	77	sigmoid cancer	1	2	268	57	20.29
Patient 17	M	75	gastric cancer	2	2	278	68	22.46
Patient 18	F	66	colon cancer	1	1	278	60	28.93
Patient 19	F	60	sigmoid cancer	1	1	275	70	22.85
Patient 20	M	57	gastric cancer	2	2	280	94	29.02
Patient 21	M	63	colon cancer	1	1	268	90	32.88
Patient 22	M	69	rectosigmoid cancer	1	2	282	80	24.25
Patient 23	M	60	gastric cancer	2	2	270	65	22.49
Patient 24	M	59	small intestine cancer	1	2	275	84	27.42
Patient 25	F	61	sigmoid cancer	1	1	247	58	26.84
Patient 26	M	73	gastric cancer	2	1	270	55	29.03
Patient 27	M	73	rectum cancer	1	2	268	60	22.25
Patient 28	F	52	sigmoid cancer	1	2	270	90	32.24
Patient 29	M	61	gastric cancer	2	2	278	69	22.77
Patient 30	M	66	sigmoid cancer	1	1	272	83	28.05
Patient 31	M	65	colon cancer	1	2	268	58	20.54
Patient 32	M	76	gastric cancer	2	1	269	80	28.72
Patient 33	F	81	gastric cancer	2	2	252	55	23.8
Patient 34	M	68	sigmoid cancer	1	2	284	80	23.62

Patient 35	M	57	gastric cancer	2	1	280	68	20.98
Patient 36	M	68	sigmoid cancer	1	1	262	200	38.2
Patient 37	F	68	colon gastric	1	2	264	200	37.28
Patient 38	F	61	rectal cancer	1	1	258	63	25.23
Patient 39	F	40	gastric cancer	2	1	268	65	23.03
Patient 40	F	61	colon cancer	1	2	268	90	32.88
Patient 41	F	67	esophagus cancer	2	2	276	60	29.36
Patient 42	M	52	esophagus cancer	2	2	268	60	22.63
Patient 43	M	59	rectosigmoid cancer	1	1	270	70	24.22
Patient 44	M	61	colon cancer	1	1	270	90	32.24
Patient 45	M	65	rectal cancer	1	1	280	225	35.49
Patient 46	F	66	rectal cancer	1	1	275	70	22.85
Patient 47	F	72	gastric cancer	2	2	260	96	37.5
Patient 48	M	82	rectal cancer	1	2	269	69	24.25
Patient 49	F	81	gastric cancer	2	1	260	60	23.43
Patient 50	M	62	rectal cancer	1	1	270	80	27.68
Patient 51	F	68	rectal cancer	1	2	268	70	24.8
Patient 52	M	55	rectal cancer	1	2	275	68	22.2
Patient 53	M	63	rectal cancer	1	2	275	205	34.28

Table S2. The results of Levene's test for the first seven PCs and the inflammatory markers. *P*-value < 0.05 shows significant difference between the control and GCRC groups.

Parameter	*P*-value
PC1	<0.001
PC2	0.113
PC3	0.552
PC4	0.436
PC5	0.148
PC6	0.386
PC7	0.847
CRP	<0.001
Neutrophil Count	0.001
Platelet Count	0.013
Hemoglobin	0.001

Table S3. The descriptive statistics for the first seven PCs and for the inflammatory markers for the two groups, Ctrl and GCRC.

Parameter	Group	Mean	Standard Deviation	Variance
PC1	Ctrl	2.264	3.747	14.038
	GCRC	− 1.068	7.278	52.963
PC2	Ctrl	− 1.154	2.954	8.726
	GCRC	0.544	4.516	20.396
PC3	Ctrl	− 1.095	2.746	7.543
	GCRC	0.519	3.261	10.635
PC4	Ctrl	0.279	2.663	7.093
	GCRC	− 0.131	3.177	10.093
PC5	Ctrl	0.797	1.615	2.609
	GCRC	− 0.376	2.379	5.626
PC6	Ctrl	− 0.377	1.049	1.100
	GCRC	0.178	1.433	2.054
PC7	Ctrl	− 0.339	1.205	1.453
	GCRC	0.160	1.130	1.278
CPR	Ctrl	− 0.021	0.087	0.008
	GCRC	0.152	0.347	0.120
Neutrophil Count	Ctrl	− 0.042	0.007	0.003
	GCRC	0.078	0.210	0.044
Platelet Count	Ctrl	− 0.011	0.062	0.004
	GCRC	0.032	0.172	0.029
Hemoglobin Level	Ctrl	0.024	0.112	0.012
	GCRC	− 0.059	0.239	0.057

Table S4. The results of the univariate data analysis for the CRP level, neutrophil count, platelet count and hemoglobin level.

	**AUC**		**Ctrl Subjects**	**GCRC Patients**
C-Reactive Protein Level	0.59	Average	1.13	2.08
		Standard deviation	0.47	1.89
		Median	1.00	1.43
Neutrophil Count	0.69	Average	3.86	5.44
		Standard deviation	0.74	2.74
		Median	3.99	4.79
Platelet Count	0.52	Average	280.6	312.7
		Standard deviation	46.25	126.4
		Median	289.0	277.0
Hemoglobin Level	0.58	Average	13.19	12.53
		Standard deviation	0.88	1.89
		Median	13.30	12.70
